# Metaheuristic-Driven Ensemble Learning for Robust Fracture Energy Prediction in FDM-Fabricated PLA Components

**DOI:** 10.3390/polym18040470

**Published:** 2026-02-12

**Authors:** Volkan Ates, Mehmet Eker, Ramazan Gungunes, Demet Zalaoglu

**Affiliations:** 1 Department of Computer Engineering, Tarsus University, Mersin 33400, Turkey; 2 Department of Mechanical Engineering, Tarsus University, Mersin 33400, Turkey; mehmeteker@tarsus.edu.tr; 3 Electrical Program, Department of Electricity and Energy, Vocational School, Kirikkale University, Kirikkale 71450, Turkey; ramazangungunes@kku.edu.tr; 4 Department of Mechanical Engineering, Osmaniye Korkut Ata University, Osmaniye 80000, Turkey; demetkayretli@osmaniye.edu.tr

**Keywords:** fused deposition modeling, polylactic acid, fracture energy, Taguchi method, grey wolf optimizer, ensemble learning, optimization, machine learning

## Abstract

Additive manufacturing (AM) has reshaped production methodologies by enabling the fabrication of complex geometries for high-performance applications. As a leading AM technique, Fused Deposition Modeling (FDM) is widely used for its versatility. However, the structural reliability of FDM-printed parts is fundamentally dictated by their mechanical performance, where impact toughness functions as a critical benchmark across demanding industrial environments. Polylactic acid (PLA) has distinguished itself as a premier biodegradable polymer, favored for its superior stiffness and processability. Nevertheless, the inherent brittleness and anisotropic behavior of FDM-printed PLA pose significant challenges, necessitating investigation of their fracture mechanics. This study firstly evaluates the impact toughness of FDM-processed PLA Izod specimens using impact tests, structured within a Taguchi design of experiments (DoE) methodology. An L27 orthogonal array was employed to investigate the influence of manufacturing parameters on impact behavior and fracture energy. Then, to achieve high-fidelity predictions from experimental data, the parametric effects were systematically investigated through an advanced machine learning framework. In the first stage, optimal prediction models were identified by evaluating five mathematical formulations hybridized with five nature-inspired optimization algorithms (GWO, SMA, GSA, FPA, and KH) across nine dataset combinations. In the second stage, these best-performing models were integrated into a metaheuristic ensemble using the GWO to perform a weighted aggregation. This hybrid ensemble methodology significantly enhanced predictive accuracy, achieving a Mean Absolute Percentage Error (MAPE) of 5.0847%, which represents a 37.3% relative improvement over the best individual base model.

## 1. Introduction

The developing additive manufacturing (AM) technology, competing with traditional and computer-aided machining methods [1], is a three-dimensional printing technology used in many fields such as automotive, aerospace, medical, and architecture [2,3,4]. There are various AM techniques such as stereolithography (SLA), laminated object manufacturing (LOM), fused deposition modeling (FDM), selective laser sintering (SLS), and Fused Granular Fabrication (FGF) [5].

The FDM method is a 3D printing method that enables the production of the desired part by depositing the molten polymer filament layer by layer on the build plate with a temperature-controlled nozzle according to the computer model [6,7,8]. The production of complex-shaped parts is widely utilized in strategic sectors [9,10], such as healthcare, aerospace, chemical, petroleum, and automotive industries, due to its advantages, including reduced production time and cost [11,12]. In addition to its advantages, the FDM method also causes disadvantages such as poor mechanical properties, low dimensional and surface quality [13,14]. The quality of parts produced by the FDM method is significantly influenced by the printing parameters. Printing parameters are generally categorized into two main groups: machine parameters and process parameters [15]. Machine parameters include nozzle diameter, nozzle temperature, build temperature and machine calibration, while the process parameters contain built orientation, air gaps, raster angle, raster width, layer thickness, contour width, infill density, infill style and feed rate [5]. In the literature, the effects of printing parameters on FDM part properties, such as thermal properties [16], izod impact strength [17], and surface quality and dimensional accuracy [18], have been investigated.

Various thermoplastics such as polylactic acid (PLA), acrylonitrile butadiene styrene (ABS), polycarbonate (PC), PC-ABS blends and polyphenylsulfone are widely used in FDM technology [19]. PLA is preferred because of its low melting point, low cost, smooth printability and lower energy requirements compared to ABS and polyamides [20]. PLA is also important because of biodegradable polymer and is suitable for biomedical applications [21]. In addition to these properties, it is also highly suitable for engineering applications due to its good mechanical properties and compostable nature [22].

Many researchers have studied the effects of different printing parameters on the mechanical properties of PLA parts printed with FDM. Gajjar et al. investigated the effect of process parameters of layer thickness, raster angle, feed rate and nozzle temperature on the tensile properties of PLA parts printed with FDM. As a result of their study, they stated that tensile strength increased with the ascending of nozzle temperature and low feed rate during the 3D printing process. They also concluded that the tensile strength increased with the decrease in layer thickness and raster angle [5]. Wang et al. investigated the effects of printing parameters of layer height (0.2 and 0.4 mm) and plate temperature (30 and 160 °C) on the Izod impact strength of PLA. In this study, they stated that it has a higher Izod impact strength due to the large number of small PLA crystals formed at the PLA interfacial printed at 160 °C temperature and 0.2 mm layer height [17]. Mishra et al. studied the effect of infill pattern and density on the Izod impact strength of printed PLA. As a result of the study done, they determined that the line pattern type with 85% fill density has the best impact energy absorbing potential among all fill ratios [23]. Ansari and Kamil analyzed the effects of printing parameters (nozzle temperature, print speed, infill density and infill pattern) on Izod impact strength, hardness and part weight of carbon fibre (CF) reinforced PLA composites with Taguchi experimental design. They also determined the most suitable printing parameters using ANOVA analysis. They found that Izod impact strength was affected by the printing speed and filler density parameters, and the maximum Izod impact strength was obtained at 100% infill density, 210 °C nozzle temperature and 80 mm/s printing speed for the grid type infill pattern. However, they found the response optimization for parameters such as maximum Izod impact strength, maximum hardness and low part weight in the grid type infill pattern with 240 °C nozzle temperature, 120 mm/s printing speed and 50% infill density [13]. Pachaur et al. optimized the printing parameters to obtain the best tensile strength in FDM printed PLA materials. They tried to determine the optimum combination of parameters using Taguchi’s L27 orthogonal array and ANOVA optimization techniques for the PLA part with maximum tensile strength [24]. Skorokhoda et al. presents the degradation of polylactide (PLA) was most effective under aerated conditions, with the fungus Penicillium chrysogenum and the bacterium Bacillus subtilis achieving the highest rates. Microbial action also altered PLA’s material properties, often increasing its crystallinity and thermal stability [25].

In this study, the experimental design strategy employed for the fabrication of PLA samples is grounded in well-established principles of robust design and statistical optimization. The Taguchi method is a widely accepted design of experiments (DOE) approach that uses orthogonal arrays to systematically investigate the influence of multiple process parameters on output responses while minimizing the total number of experimental runs required. By selecting appropriate orthogonal arrays, balanced and statistically interpretable experiments can be conducted even when multiple factors and levels are involved, thereby enhancing experimental efficiency without compromising analytical validity [26].

Numerous studies in the additive manufacturing and polymer processing literature have successfully applied the Taguchi method to optimize PLA-based samples, validating its suitability for multi-parameter experimental investigation. For example, investigations into the mechanical properties of PLA components produced via FDM have utilized Taguchi orthogonal arrays to determine optimal process settings for tensile and compressive performance. In one study, the influence of layer thickness, infill density, printing speed, and other parameters on the mechanical behavior of PLA was evaluated using a Taguchi DOE framework [27], and another similarly structured work analyzed tensile properties of FDM-printed PLA specimens through Taguchi statistical techniques to quantify factor significance [28]. Further evidence of the method’s applicability to PLA research is provided in studies that employed Taguchi designs to assess dimensional accuracy and surface quality of thin-walled PLA prints at multiple factor levels [29].

The effects of additive manufacturing parameters on the impact resistance of Izod specimens manufactured via the FDM method have been systematically investigated using a Taguchi L27 orthogonal array methodology, where the effectiveness of design parameters on fracture energies was determined. Given that the fracture mechanics of FDM-printed components are governed by inherently nonlinear, multivariate, and interdependent mechanisms, traditional linear models often fail to generalize across diverse manufacturing conditions. To address these constraints, a two-stage machine learning-based prediction system has been developed. The significance of this study lies in the development of a novel two-stage hybrid ensemble learning framework designed to address the nonlinear, multivariate, and interdependent mechanisms governing the fracture behavior of FDM components.

During the initial stage, optimal prediction models were identified by evaluating 5 distinct mathematical formulations (Linear, Exponential, Power, Quadratic, and Semi-Quadratic) in conjunction with 5 nature-inspired optimization algorithms (Grey Wolf Optimizer (GWO), Slime Mould Algorithm (SMA), Gravitational Search Algorithm (GSA), Flower Pollination Algorithm (FPA), and Krill Herd (KH)), across 9 unique dataset combinations. In the subsequent stage, these optimal models were integrated using an ensemble methodology, further optimized via the Grey Wolf Optimizer (GWO) algorithm, to yield a consolidated model with enhanced predictive performance. This study addresses a critical research gap arising from the inherent brittleness and anisotropic behavior of FDM-fabricated PLA components, whose fracture mechanisms are governed by complex, nonlinear, and interdependent processes that conventional linear models fail to adequately capture. Therefore, this study aims to contribute to existing literature by presenting an alternative optimization approach using machine learning techniques to determine the optimum printing parameters in FDM manufacturing processes.

## 2. Materials and Methods

In Fused Deposition Modeling (FDM) processes, the quality and mechanical integrity of the final product are influenced by a multitude of variables, broadly categorized into machine parameters such as nozzle and bed temperatures and process parameters, including infill density, print orientation, raster angle, printing speed, and layer thickness.

The raster angle is defined by the infill orientation or the printing direction of the filament during the FDM process, where 0 is perpendicular to the loading direction and 90 is parallel to the loading direction [30]. The term “print orientation” used in the study refers to the knitting pattern used in PLA production. The grid pattern uses a simple, cubic lattice of intersecting lines, suitable for large flat surfaces but prone to nozzle clogging from overlaps. The octet pattern creates internal pyramid-like structures that provide excellent support for horizontal surfaces, allowing for better top layers without increasing density. The gyroid pattern consists of complex, continuous wavy curves, offering nearly uniform strength in all horizontal directions and good performance with flexible materials, though it takes longer to print [31]. The nozzle temperature, typically set between 190 °C and 220 °C, ensures the filament melts properly for smooth extrusion; the bed temperature, commonly maintained at 50 °C to 60 °C, promotes strong first-layer adhesion and minimizes warping; and the layer thickness (or height), which defines the vertical resolution on the *Z*-axis and directly influences the print’s surface finish, detail, and total printing time.

Wickramasinghe et al. explains how multiple process parameters—including print orientation (OR), raster angle (RA), nozzle temperature (NT °C), bed temperature (BT °C), and layer thickness (LT mm)—jointly control the microstructural evolution of printed parts in fused deposition modeling (FDM) is supported by extensive literature demonstrating their interdependent effects on key microstructural features such as interlayer bonding, porosity, void formation, anisotropy, crystallinity, and defect distribution. Comprehensive reviews and experimental studies show that print orientation and raster angle govern filament alignment, load paths, and anisotropic microstructure (e.g., promoting delamination in weak orientations or fiber-like fracture in aligned ones), while nozzle and bed temperatures regulate melt viscosity, interlayer diffusion, residual stresses, warping, shrinkage, and phase evolution (e.g., higher temperatures enhance bonding but risk degradation). Layer thickness modulates interface density, cooling rates, and bond quality, influencing pore size, homogeneity, and overall microstructural integrity, as evidenced through SEM fracture analysis and quantitative pore metrics. These parameters interact synergistically, with changes in one often amplifying or mitigating the effects of others on microstructure and resulting mechanical performance [32].

The mechanical response of FDM-printed components is governed by these variables, which are inherently multivariate and interdependent in nature. Due to these complex effects, a systematic analysis is required to understand how the interaction of variables affects material properties. To effectively manage this complexity, Design of Experiment (DoE) techniques are utilized to evaluate the influence of multiple independent factors on experimental responses. A prominent DoE approach is the Taguchi method, which enables the development of high-quality products and processes with a minimum number of experiments and reduced costs, while providing a powerful framework for identifying both the main effects and interaction effects of critical process parameters [33].

### Taguchi Experimental Design

To systematically evaluate the FDM process, five critical printing parameters, which are print orientation, raster angle, layer thickness, nozzle temperature, and bed temperature, were investigated at three distinct levels as given in Table 1. Each selected level and specific combinations were strategically chosen to enable the production of specimens with distinct mechanical and microstructural properties. For this objective, the Taguchi method was applied to determine the effectiveness of these design parameters on fracture energies. The method identified the L27 orthogonal array as the most appropriate experimental matrix for the given number of factors and their levels. This matrix is particularly powerful for identifying the main effects and interaction effects of process parameters on the response variable, making it ideal for robust design optimization in engineering fields with limited experimental resources. The L27 orthogonal array, designed to guide the fabrication of the 27 unique experimental combinations, is presented in Table 2.

In this study, Izod impact test specimens were fabricated via the FDM in accordance with the ASTM D256 standard, which governs the test methods for determining the Izod pendulum impact resistance of plastics. The specimen geometries were designed in CAD software (5.1) following the configurations set by the Taguchi L27 experimental matrix. These three-dimensional models were processed using Creality Print 5.1 software to generate 27 unique gcode files. Manufacturing was carried out on a Creality Ender 3 S1 Pro 3D (Shenzhen, China) printer using PLA filament. To ensure statistical reliability, three replicates were produced for each of the specimen designs, resulting in a total of 81 specimens. Impact testing was performed using an ALSA 450 CE testing (Istanbul, Turkey) unit to measure the fracture energy. Finally, the average fracture energy for each experimental run was determined by calculating the arithmetic mean of the results obtained from the three replicates.

## 3. Mathematical Modelling of Machine Learning Algorithm

While the Taguchi method provides a foundational understanding of parameter effects on fracture energies, a more advanced computational approach is required because FDM printing parameters interact with each other and influence the material behavior in complex ways. Understanding the complex interplay between production variables is essential for optimizing the fracture behavior of PLA components. To accurately capture these multi-parameter interactions, this study utilizes a two-stage hybrid ensemble framework optimized via the Nature-Inspired optimization algorithms.

### 3.1. Theoretical Framework

Accurately predicting the fracture energy (E_f_, Jm^−2^) of components fabricated by FDM remains one of the central challenges in additive manufacturing research due to the inherently nonlinear, multivariate, and interdependent nature of the process. Multiple process parameters—including print orientation (OR), raster angle (RA), nozzle temperature (NT °C), bed temperature (BT °C), and layer thickness (LT mm)—jointly control the microstructural evolution of printed parts. These parameters directly influence interlayer adhesion, void morphology, crystallinity development, and residual stress distribution, all of which determine the mechanical response during crack initiation and propagation [34,35].

Interdependence among process variables means that a change in one parameter can modify the influence of others. For example, increasing NT enhances molecular mobility and diffusion, strengthening interlayer bonds but simultaneously altering the role of RA in fracture behavior. Similarly, changes in OR modify anisotropy and affect heat conduction pathways, influencing crystallization kinetics and residual stress fields [36]. These coupled effects highlight the need for predictive models capable of representing multi-parameter interactions, rather than relying solely on additive effects.

Mathematically, the fracture energy can be represented as:

(1) $$E_{f}=\;f(OR,RA,NT,BT,LT;w)$$

where w = [w_0_ + w_1_,…,w_n_] is the vector of model coefficients estimated through optimization.

**Figure 1:**
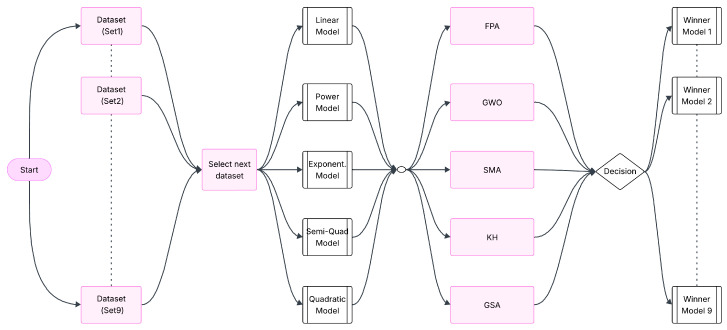
Flowchart of the Optimization phase of empirical mathematical models.

Five model formulations—linear, power-law, exponential, semi-quadratic, and quadratic—were employed, each capturing distinct physical phenomena. Linear models provide first-order sensitivity analysis, while power-law and exponential models describe multiplicative scaling and regime transitions. Quadratic models are especially suited for capturing curvature and second-order interactions [37,38]. In this study, three datasets that are theoretically equivalent were utilized. To reduce the influence of variability introduced by measurement conditions, the datasets were combined in a pairwise manner to generate multiple training and testing configurations. This combinatorial approach ensured that each dataset was alternately used for model training and validation. Figure 1 presents the optimization process of empirical mathematical models using nature-inspired optimization algorithms based on these dataset combinations.

These approaches are crucial not only for theoretical understanding but also for industrial applications where fracture performance dictates the reliability of aerospace, biomedical, marine and automotive components. Furthermore, while this framework is tailored for thermo-plastic polymers under standard FDM conditions, future extensions could address fiber-reinforced composites and multi-material systems with more complex fracture mechanisms.

The optimization phase involved evaluating multiple dataset combinations, with the predictive performance of each algorithm assessed independently to ensure a fair and unbiased comparison. Model accuracy was quantified using standard performance metrics widely adopted in the literature, including Mean Absolute Error (MAE), Mean Absolute Percentage Error (MAPE), Mean Squared Error (MSE), Root Mean Squared Error (RMSE), and the coefficient of determination (R^2^). These metrics enabled a systematic comparison between predicted and observed values, facilitating the identification of the most robust algorithm for each dataset configuration. $y_{i}$ is the real Fracture Energy, ${\hat{y}}_{i}$ is the predicted and $\bar{y_{i}}$ is the mean value. Coefficients are determined by minimizing loss functions such as MAPE or mean squared error (MSE), subject to model constraints.

(2) $$MAPE=\frac{100}{N}\sum_{i=1}^{N}\left|\frac{y_{i}-{\hat{y}}_{i}}{y_{i}}\right|$$

(3) $$MSE=\frac{1}{N}\sum_{i=1}^{N}{\left(y_{i}-{\hat{y}}_{i}\right)}^{2}$$

(4) $$MAE=\frac{1}{N}\sum_{i=1}^{N}\left|y_{i}-{\hat{y}}_{i}\right|$$

(5) $$RMSE=\sqrt{\frac{1}{N}\sum_{i=1}^{N}{\left(y_{i}-{\hat{y}}_{i}\right)}^{2}}$$

(6) $$R^{2}=1-\sum_{i=1}^{N}\frac{{\left(y_{i}-{\hat{y}}_{i}\right)}^{2}}{{\left(y_{i}-\bar{y_{i}}\right)}^{2}}$$

### 3.2. Model Structures and Physical Interpretations

Empirical equations serve to provide a compact and interpretable framework for simulating and optimizing FDM processes. By fitting equation parameters to experimental data, these models can predict how adjustments to processing variables—such as nozzle temperature or layer time—influence critical material properties like fracture toughness. This predictive capability aids in the design of stronger 3D-printed components, reducing reliance on iterative experimental methods. Empirical models are particularly valuable for capturing nonlinear “threshold” behaviors, such as a sudden improvement in material performance beyond a specific temperature, which linear models often fail to represent. Consequently, they constitute a practical tool for applied materials science and additive manufacturing research. This approach aligns with principles discussed in related work on additively manufactured polymers. For instance, Patel et al. investigated fracture energy and scaling behaviors in polymers fabricated via two-photon lithography. While their study did not focus on FDM specifically, the underlying principles concerning thermal processing effects, interlayer adhesion, and nonlinear fracture mechanics show significant overlap, reinforcing the broader applicability of empirical modeling in additive manufacturing [39].

#### 3.2.1. Linear Model

The linear model assumes that each process parameter contributes independently and additively to fracture energy. Its primary advantage lies in interpretability and the ability to estimate first-order sensitivities, providing initial insights into the relative importance of variables. However, its simplicity also constitutes its main limitation, as it neglects cross-parameter interactions, nonlinear coupling effects, and threshold phenomena, all of which are common in polymer crystallization, anisotropic stress fields, and layer-fusion kinetics [36]. Consequently, while linear modeling is suitable for preliminary analysis and feature screening, it often fails to generalize beyond narrow process windows.

(7) $${\hat{E}}_{f}=w_{0}+w_{1}OR+w_{2}RA+w_{3}NT+w_{4}BT+w_{5}LT$$

In the expression above, ${\hat{E}}_{f}\;$ is the predicted fracture energy; OR, RA, NT, BT, and LT are the process variables; w_0_ is the intercept (baseline energy); and w_1_…w_5_ are regression coefficients quantifying the linear sensitivity of ${\hat{E}}_{f}\;$ to each variable under the additivity assumption.

#### 3.2.2. Power-Law Model

The power-law model encodes scaling behavior between processing and properties that is ubiquitous in thermally driven polymer processes. It is particularly pertinent in FDM where increases in NT or LT often yield non-proportional gains in interlayer bonding and energy dissipation due to enhanced chain mobility and diffusion. Here, ${\hat{E}}_{f}$ is the predicted fracture energy; $x_{k}\in \left\{OR,RA,NT,BT,LT\right\}$ denotes the kth process variable; a_0_ is the baseline term; a_k_ is the amplitude (how strongly ${\hat{E}}_{f}\;$ responds to x_k_); and b_k_ is the scaling exponent capturing the type of nonlinearity: b_k_ > 1 indicates super-linear synergy (e.g., rapid toughness gains past a processing threshold), while 0 < b_k_ < 1 captures diminishing returns typical of diffusion-limited or saturation regimes. Compared with the linear model, the power-law form better reflects kinetics-driven effects without explicitly invoking interaction terms [36].

(8) $${\hat{E}}_{f}=a_{0}+\sum_{k=1}^{5}a_{k}x_{k}^{b_{k}},\;b_{k}>0$$

#### 3.2.3. Exponential Model

The exponential model targets threshold and regime-transition phenomena, such as the abrupt rise in interlayer adhesion when NT nears the polymer’s *T_g_*, or changes associated with crystallization onset. In this formulation, c_0_ denotes the baseline energy, c_k_ the magnitude of each exponential contribution, and d_k_ the growth rate governing how quickly ${\hat{E}}_{f}\;$ escalates with the variable $x_{k}\in \left\{OR,RA,NT,BT,LT\right\}$. Because $e^{d_{k}x_{k}}$ grows rapidly, this model captures activation-like responses that linear or power-law forms often miss but also requires careful variable scaling to avoid numerical dominance by any single term. Physically, it is well-suited for sharp, thermally mediated changes in bonding and fracture mechanisms [34,35].

(9) $${\hat{E}}_{f}=c_{0}+\sum_{k=1}^{5}c_{k}e^{d_{k}x_{k}}$$

#### 3.2.4. Semi-Quadratic Model

The semi-quadratic model introduces square-root responses to represent sub-linear gains frequently observed once diffusion lengths or crystallization levels begin to saturate. Here, $\gamma_{0}$ is the intercept; $\gamma_{k}$ weights the direct $\sqrt{x_{k}}$ effect of each variable $x_{k}\in \left\{OR,RA,NT,BT,LT\right\};{\;\eta }_{k}$ weights an augmented root term scaled by $d_{k}$, providing extra flexibility to fit early-stage sensitivity while avoiding the full complexity of quadratic interactions. Conceptually, the square-root dependency encodes diminishing marginal improvements in ${\hat{E}}_{f}\;$ as processing increases—useful when early parameter changes strongly improve bonding, but subsequent increases yield progressively smaller toughness gains.

(10) $${\hat{E}}_{f}=\gamma_{0}+\sum_{k=1}^{5}\gamma_{k}\sqrt{x_{k}}+\sum_{k=1}^{5}\eta_{k}\sqrt{d_{k}x_{k}}$$

#### 3.2.5. Quadratic Model

The quadratic model provides a second-order response surface that captures both curvature and pairwise interactions, making it the most expressive of the considered forms for FDM fracture mechanics. In the equation, $\beta_{0}\;$ is the intercept; $\beta_{k}\;$ quantify linear effects of each $x_{k}\in \left\{OR,RA,NT,BT,LT\right\};\;\beta_{kk}$ code self-nonlinearity (how the effect of $x_{k}\;$ changes with its magnitude); and $\beta_{kj}$ describe cross-effects between parameters $x_{k}$ and $x_{j}$. Practically, this model aligns with response-surface methodology, often delivering the highest predictive accuracy when designed with adequate data coverage, while requiring care to control multicollinearity and overfitting [36].

(11) $${\hat{E}}_{f}=\beta_{0}+\sum_{k=1}^{5}\beta_{k}x_{k}+\sum_{k=1}^{5}\beta_{kk}x_{k}^{2}+\sum_{k=1}^{5}\sum_{j\neq k}\beta_{kj}x_{k}x_{j}$$

## 4. Dataset Preparation and Experimental Design

Three datasets (27 specimens each) were generated using a full factorial design with parameters varied at three levels (low, medium, high). Each dataset represented a distinct batch, intentionally incorporating variability from material feedstock, calibration, and environmental conditions. Fracture energy was measured via standardized impact testing. To evaluate model generalization, nine train–test configurations were created using cross-batch validation, where models trained on one or more batches were tested on others. This method rigorously assesses robustness beyond interpolation and is widely used in materials informatics [36].

### 4.1. Train-Test Strategy and Cross-Batch Validation

To rigorously evaluate the generalizability of the predictive models, a comprehensive cross-batch validation scheme was implemented. Nine distinct train–test configurations were constructed, systematically alternating the datasets as training and testing sets as given in Table 3:

This approach, analogous to cross-domain or cross-batch validation in material informatics, enables a more stringent assessment of model robustness [36,37,38]. Unlike conventional k-fold methods, it explicitly evaluates the capacity of models to extrapolate across unseen manufacturing conditions—a critical requirement for predictive deployment in real-world additive manufacturing scenarios.

### 4.2. Metaheuristic Optimization

In the present study, five well-established metaheuristic optimization algorithms were rigorously compared under identical experimental conditions to ensure equitable performance assessment. The standardized benchmark configuration employed a population size of 50 search agents, a maximum of 1000 iterations, and a search space bounded within the interval [−10, 10]. Notably, SMA, GWO, and GSA exhibit minimal requirements for additional parameter tuning, thereby conferring greater robustness and ease of implementation, whereas FPA necessitates judicious calibration of its switch probability (set to 0.8 in this study, a commonly adopted value in the original formulation and many benchmark investigations to favor global pollination while retaining sufficient local search capability). The source codes of the Nature-Inspired Optimization algorithms were obtained from the official MATLAB website and adapted to the nature of the problems.

All simulations and optimization routines were implemented in MATLAB (R2023b) using in-house codes developed by the authors. Computations were performed on a workstation equipped with an Intel Core i9-10900KF processor (10 cores, 20 threads, up to 5.3 GHz), 64 GB DDR4 RAM (3200 MHz), and an NVIDIA RTX 2070 SUPER GPU (8 GB GDDR6), running Windows 10 Pro (64-bit). The computational cost was moderate and compatible with iterative parametric studies typical of experimental additive manufacturing research.

#### 4.2.1. Grey Wolf Optimizer (GWO)

The GWO, inspired by the leadership hierarchy and hunting strategy of grey wolves [40], was employed for coefficient estimation. Candidate solutions (α,β,δ) iteratively guide the search, balancing exploration (global search) and exploitation (local search) by gradually decreasing the control parameter A.

Recent advancements, including multi-strategy GWO [41] and scale-free topology enhancements [42], significantly improve convergence speed and solution quality, particularly in high-dimensional, nonlinear optimization spaces. These improvements strengthen parameter estimation and enhance model robustness. Due to these advantages, this methodology has also found a wide range of applications in current literature [43].

For a given candidate solution (wolf position) X(t) at iteration t, its position is updated relative to the top three solutions (α,β,δ) as follows:

(12) $$D_{\alpha }=\left|C_{1}X_{\alpha }-X\left(t\right)\right|\;D_{\beta }=\left|C_{2}X_{\beta }-X(t)\right|\;D_{\delta }=\left|C_{3}X_{\delta }-X(t)\right|\;X_{1}=X_{\alpha }-A_{1}D_{\alpha }\;X_{2}=X_{\beta }-A_{2}D_{\beta }\;X_{3}=X_{\delta }-A_{3}D_{\delta }\;X(t+1)=\frac{X_{1}+X_{2}+X_{3}}{3}$$

Here, $A_{i}$ and $C_{i}\;$ are adaptive coefficient vectors controlling convergence dynamics. This update mechanism guides the search toward the optimum while preserving population diversity. The optimization process using the GWO follows a structured sequence of computational steps designed to balance global exploration and local exploitation within the solution space. The procedure can be described as follows:

**Initialization:** A population of grey wolves, each representing a candidate solution vector (i.e., a set of model coefficients), is randomly initialized within predefined search boundaries across the solution space. This ensures a diverse starting distribution and enhances the algorithm’s exploratory capacity.

**Fitness Evaluation:** The fitness of each candidate solution is evaluated based on a predefined objective function- typically a model performance metric such as MAPE or MSE. This step quantifies how well each solution approximates the target fracture energy behavior.

**Leadership Hierarchy Formation:** The three best-performing solutions in the current population are identified and designated as the α, β, and δ wolves, representing the leading, secondary, and tertiary solutions, respectively. These leaders guide the optimization process and influence the position updates of the remaining population (ω wolves).

**Position Updating:** Each wolf updates its position in the solution space by dynamically adjusting its distance and direction relative to the α, β, and δ leaders. This step mathematically simulates the encircling and hunting behavior observed in grey wolf packs, progressively moving the population toward regions of higher fitness.

**Convergence Phase:** The exploration–exploitation balance is adaptively controlled by gradually reducing the control coefficient A from 2 to 0 throughout the iterations. This mechanism transitions the search process from broad exploration in the early stages to refined exploitation near promising optima in later stages.

**Termination:** The optimization process terminates when a predefined stopping criterion is satisfied-either when the maximum number of iterations is reached or when the improvement in solution quality falls below a convergence threshold. The best solution identified at this stage (typically the α wolf) is returned as the final optimized set of model coefficients.

#### 4.2.2. Gravitational Search Algorithm (GSA)

GSA is a population-based metaheuristic optimization technique inspired by Newton’s laws of gravity and motion. The algorithm represents candidate solutions as agents characterized by varying masses, which interact through gravitational forces within the search space. The position of each agent corresponds to a potential solution vector, while its mass is determined by the objective function value (fitness). Superior solutions are assigned greater masses, whereas inferior solutions receive smaller masses. Consequently, agents with heavier masses exert stronger gravitational attraction, influencing the movement of other agents toward more promising regions of the search space [44].

Force values are calculated by formula:

(13) $$F_{ij}=G(t)\frac{M_{i}M_{j}}{R_{ij}+\;\varepsilon }\left(x_{i}-\;x_{j}\right)$$

G(t) is gravitational constant (decreases over time), $M_{i}{,\;M}_{j\;}$ are mass values, $R_{ij}$ Euclidean distance, $x_{i},\;x_{j}$ agent position and ε  correction constant.

Acceleration $a_{i}$, movement velocity $v_{i}\;$ and position changes $x_{i}$ are calculated by:

(14) $$a_{i}=\frac{F_{i}}{M_{i}}$$

(15) $$v_{i}\left(t+1\right)=rand\cdot v_{i}\left(t\right)\cdot a_{i}\left(t\right)$$

(16) $$x_{i}\left(t+1\right)=\left(t\right)\cdot v_{i}\left(t+1\right)$$

GSA primary innovation lies in modelling candidate solutions as interacting masses, where solution quality dynamically determines gravitational influence, enabling multiple high-quality solutions to guide the search process simultaneously. This distributed leadership mechanism reduces the risk of premature convergence compared to single-leader strategies. Furthermore, the use of a time-decreasing gravitational constant provides an adaptive balance between global exploration and local exploitation, which was not explicitly incorporated in earlier population-based algorithms.

#### 4.2.3. Krill Herd Algorithm (KH)

The Krill Herd Algorithm (KH) constitutes a swarm intelligence-based metaheuristic optimization approach, drawing inspiration from the herding behavior exhibited by krill swarms in marine environments. In natural settings, krill individuals aggregate into extensive, dynamic swarms to improve survival probabilities through diminished predation vulnerability and enhanced foraging efficacy. Such aggregations display intricate, emergent motion patterns arising from local inter-individual interactions, responses to environmental cues, and adaptive collective coordination. The KHA formalizes these biological processes through mathematical modeling to direct the optimization search, wherein each krill individual corresponds to a potential solution, and the population as a whole navigates the solution space via collaborative and adaptive locomotion mechanisms [45].

In the ocean, krill form dense swarms to avoid predators (potential enemies), increase food intake and improve survival. Krill Herd movement is governed by neighbor interactions, foraging activity and random positioning.

Position of the individuals are calculated by:

(17) $$∆X_{i}\left(t\right)=N_{i}+\;F_{i}+D_{i}$$

(18) $$N_{i}=N_{max}(\alpha_{i}+\beta_{i})$$

(19) $$F_{i}=V_{f}(\gamma_{i}+\delta_{i})$$

(20) $$D_{i}=D_{max}\cdot rand$$

where in formulas $∆X_{i}\left(t\right)$ is the position change, $N_{i}$ is motion induced by other krill, $F_{i}$ is foraging motion, $D_{i}$ is random diffusion, $\alpha_{i}$ is the local effect of the neighbor Krill, $\beta_{i}$ best Krill effect, $V_{f}$ is the foraging speed, $\gamma_{i}$ is the food location effect, and $\delta_{i}$ is the stored previous best position.

Next position of the individual Krill is calculated by:

(21) $$X_{i}\left(t+1\right)=X_{i}\left(t\right)+\;∆t(N_{i}+F_{i}+D_{i})$$

KH integrates multiple behavioral components to achieve a more realistic and adaptive search process. Its hybrid structure, which incorporates evolutionary operators such as crossover and mutation, further enhances population diversity and global exploration capability. This multi-mechanism design distinguishes KH from existing metaheuristics and contributes to its improved convergence stability and robustness against premature stagnation.

#### 4.2.4. Slime Mould Algorithm (SMA)

The Slime Mould Algorithm (SMA), introduced by Li et al. (2020) [46], is a population-based bio-inspired metaheuristic optimization method inspired by the foraging behavior of the true slime mould Physarum polycephalum. This single-celled amoeboid organism exhibits sophisticated collective intelligence through dynamic vein-like networks that expand, shrink, and restructure in response to nutrient availability, transporting protoplasm via rhythmic shuttle-streaming oscillations. High-quality paths are reinforced by positive feedback (increased flow and thickness), while inefficient routes decay via negative feedback, enabling decentralized solutions to complex problems such as shortest-path routing and network optimization—capabilities demonstrated in laboratory experiments involving mazes and minimal spanning networks [46].

Slime moulds are single-celled organisms that exhibit intelligent foraging behavior based on forming dynamic vein-like networks. They adjust thickness of the veins according to food quality and distance. Each cell is a candidate solution, and the positions of the cells are updated based on the weights and oscillatory movement.

Position update and adaptive weights are calculated by:

(22) $$X_{i}\left(t+1\right)=\left\{\begin{array}{c} \begin{array}{c} X_{best}\left(t\right)\;v_{b}\;\cdot \;\left(W_{i}\;\cdot \;X_{A}-\;X_{B}\right),\;\;\;r<p \end{array}\\ X_{i}\left(t\right)\;\cdot \;v_{c},\;\;\;\;\;\;\;\;\;\;\;\;\;\;\;\;\;\;\;\;r\geq p \end{array}\right.$$

(23) $$W_{i}=\left\{\begin{array}{c} \begin{array}{c} 1+rand\;\cdot log\;(\frac{bF-f_{i}}{bF-wF}+1),\;\;\;i\leq \frac{N}{2} \end{array}\\ 1-rand\;\cdot log\;(\frac{bF-f_{i}}{bF-wF}+1),\;\;\;i>\frac{N}{2} \end{array}\right.$$

where in formulas $X_{best}$ is the best solution, $X_{A}\;\;and\;\;X_{B}$ are random solutions, $W_{i}$ is adaptive weight, $v_{b}\;\;\mathrm{and}\;\;v_{c}$ are oscillation parameters, p  is a probability parameter, r is a random number, bF is the best fitness, wF is the worst fitness and $f_{i}$ is the agent fitness.

The key innovation of SMA lies in the use of fitness-driven adaptive weighting combined with positive and negative feedback mechanisms, which dynamically reinforce high-quality solutions while suppressing inferior ones. Unlike conventional swarm algorithms that rely on velocity-based updates or fixed leadership structures, SMA employs oscillation-based position adaptation, allowing the search process to self-regulate between exploration and exploitation. This biologically grounded feedback learning strategy distinguishes SMA from existing metaheuristics and enhances its robustness against premature convergence.

#### 4.2.5. Flower Pollination Algorithm (FPA)

The Flower Pollination Algorithm (FPA), proposed by Xin-She Yang in 2012, is a nature-inspired population-based metaheuristic optimization algorithm that mathematically models the pollination process of flowering plants. In FPA, each flower (or pollen gamete) represents a candidate solution in the search space, and solution evolution occurs through simulated pollination over iterations [47].

The algorithm is founded on four idealized rules:

1. Biotic cross-pollination and abiotic pollination are modeled as global pollination, involving long-distance pollen transfer via pollinators (for biotic) or wind/diffusion (for abiotic), implemented using Lévy flight-based random walks to explore distant regions and avoid local optima.
2. Abiotic self-pollination and local biotic pollination among nearby flowers are modeled as local pollination, using small random perturbations for exploitation in promising areas.
3. Flower constancy, where pollinators prefer certain flower species, is approximated by a proportionality in reproduction probability related to solution similarity or fitness difference.
4. The switch between global and local pollination is governed by a probability parameter *p* ∈ [0, 1], often biased toward local search (e.g., *p* = 0.8 in many studies) due to proximity factors.

The exploration phase of the algorithm is based on Levy flight diversity, which is formulated as follows:

(24) $$X_{i}\left(t+1\right)=X_{i}\left(t\right)+\;L(g^{*}-\;X_{i}\left(t\right))$$

(25) $$L\sim \;\frac{\lambda \Gamma (\lambda )sin(\frac{\pi \lambda }{2})}{\pi }\;\cdot \;\frac{1}{s^{1+\lambda }}$$

The exploitation phase of the algorithm is formulated as follows

(26) $$X_{i}\left(t+1\right)=X_{i}\left(t\right)+\varepsilon (X_{j}\left(t\right)-X_{k}\left(t\right))$$

where in formulas $g^{*}$ is the global best solution, L  is the Levy step size, λ is the stability parameter in the Levy index, and ε is a random number drawn from a uniform distribution acts as a scaling factor.

### 4.3. Ensemble Methodology

Ensemble learning refers to a class of methods in supervised machine learning that integrate multiple base learners to improve predictive performance. Each base learner is trained on labeled data to construct a model capable of generalizing to unseen examples. By combining the outputs of diverse learning algorithms, such as decision trees, neural networks, and regression models, ensemble methods aim to reduce individual model errors through mutual compensation. Consequently, this collaborative learning framework enhances robustness and accuracy compared to single-model approaches [48].

The fundamental idea of ensemble learning is to employ multiple learners and combine their predictions. The core theoretical assumption is that if a committee of M models exhibits uncorrelated errors, the average error can be reduced by a factor of M through simple averaging. In practice, however, individual model errors are typically highly correlated, leading to generally small error reduction. Nevertheless, through Cauchy’s inequality, it can be proven that the expected committee error will not exceed the expected error of the constituent models [49]. In a Bayesian context, the ensemble task often utilizes Bayesian Model Averaging (BMA), where each model’s prediction is weighted by its posterior probability [50]. BMA assumes a single model generated the entire data set, contrasting with standard ensemble methods that assume different data points may be generated by different models [50].

Ensemble learning is based on the principle of diversity, where combining models with different strengths can improve prediction accuracy. If all models make the same mistakes, the ensemble provides no benefit. Traditional methods such as majority voting and weighted averaging assume that most predictions are correct, but this assumption may fail when minority models possess specialized knowledge and higher confidence. In such cases, correct predictions can be overwhelmed by incorrect majority votes, reducing overall performance. To address this issue, this study combines diverse nature-inspired algorithms with different empirical prediction models and assigns greater weight to the best-performing structures, allowing them to work together effectively as a unified system [51].

The literature review spans over twenty years, beginning with early discussions on strategies for teaching layered neural network classification tasks by Wittner and Denker [52]. Schapire introduced boosting [50], a meta-algorithm that builds a succession of models iteratively by assigning higher weight to misclassified points. Boosting is considered the most widely used ensemble method and one of the most powerful learning ideas. The stochastic process called stochastic discrimination (SD), introduced by Kleinberg [53]. Wolpert introduced stacking, which minimizes the generalization error rate using a meta learner trained on the outputs of base learners [54]. Stacking is often used to combine models of different types, distinguishing it from bagging and boosting. Early work by Xu et al. explored methods for combining multiple classifiers (e.g., voting, Bayesian formalism, and Dempster-Shafer formalism) for applications like handwriting recognition [55]. They recommended the Dempster-Shafer formalism for its ability to obtain high recognition and reliability rates simultaneously and robustly. Breiman introduced Bagging (bootstrap aggregation), which creates multiple training sets by sampling with replacement [56]. Bagging is only effective when applied to unstable non-linear models. The most popular boosting algorithm, AdaBoost (adaptive boosting), was introduced by Freund and Schapire [57].

**Figure 2:**
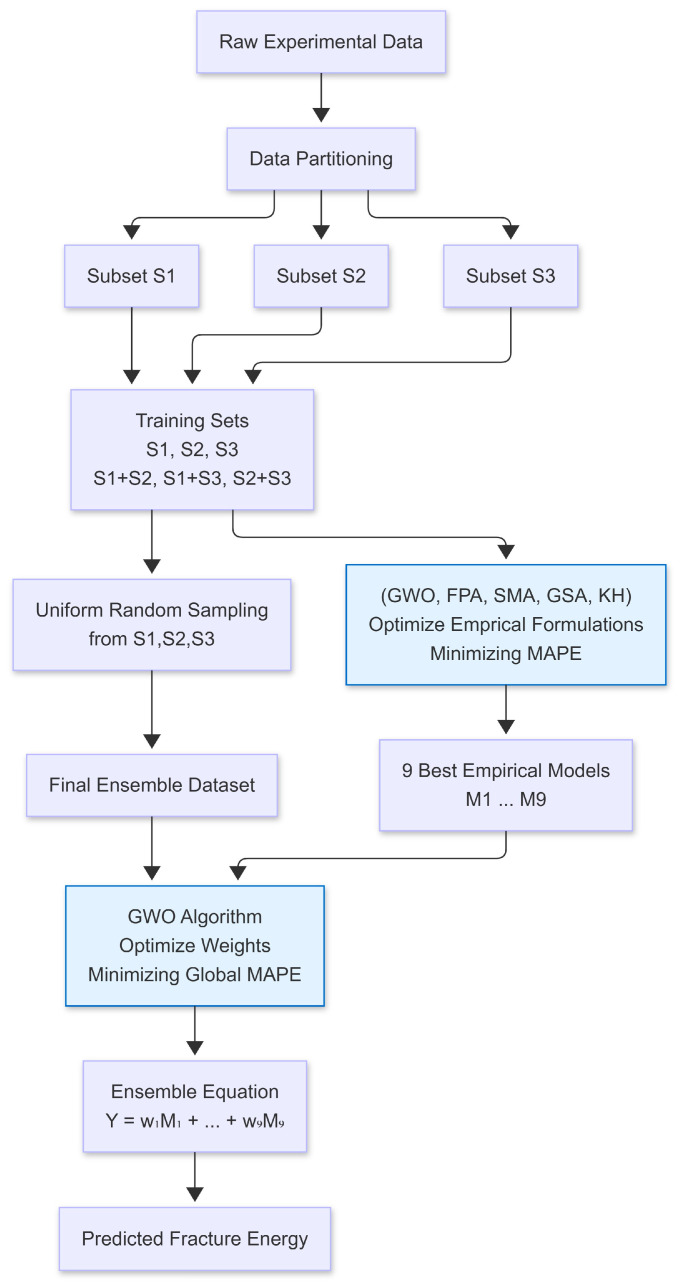
Two-Phase GWO Ensemble Framework for Fracture Energy Prediction.

This study proposes a Two-Stage Meta-Heuristic Ensemble Framework designed to enhance the predictive accuracy of fracture energy (Gf) under limited data constraints. The methodology employs a dual-layer application of GWO. In the first phase, GWO is utilized to calibrate the parameters of nine distinct empirical base learners across varied data partitions (S1, S2, S3 and their combinations), ensuring diversity in the feature space. In the second phase, a stochastic ensemble strategy is applied: a validation dataset is generated via stratified uniform random sampling, and a secondary GWO loop optimizes the weight coefficients of the base learners. This approach effectively mitigates the risk of overfitting and leverages the strengths of individual constitutive models to minimize the global MAPE. The two-stage prediction model and strategy used in the study are shown in Figure 2.

**Phase I: Data Stratification and Base Learner Calibration**

To capture the heterogeneity of the material behavior, the raw experimental dataset (N = 27) was initially stratified into three distinct subsets (S_1_, S_2_, S_3_). To foster model diversity, six training combinations were derived from these subsets (e.g., S_1_, S_1_ ∪ S_2_, etc.). For each combination, a specific empirical mathematical model was selected. The GWO algorithm was employed as the parameter estimation technique, utilizing a fitness function defined by the minimization of the MAPE for the respective training set. This resulted in six optimized ‘Base Learners’ (M_1_ … M_9_), each specialized in different regions of the data topology.

**Phase II: Stochastic Weight Optimization (Stacked Generalization)**

The second phase involved the construction of a weighted ensemble model. To ensure the robustness of the ensemble weights and prevent ‘double-dipping’ (data leakage), a representative validation dataset was constructed using Uniform Random Sampling from the primary partitions. Given the partitioning of the original data into subsets S_1_, S_2_ and S_3,_ the validation set was generated by uniformly drawing samples from the union of these partitions. This stochastic approach simulates a “Blind Test” scenario. By presenting the ensemble with a randomized mix of data points from the entire experimental domain, the optimization algorithm is forced to prioritize models that generalize well across the entire feature space, rather than models that simply overfit a specific local partition (e.g., S_1_). This stochastic sub-sampling technique allows for the simulation of unseen data distributions. The final predictive model is defined as a linear combination of the nine base learners. A secondary instance of the GWO algorithm was deployed to solve the global optimization problem: determining the optimal weight vector that minimizes the divergence between the ensemble prediction and the sampled experimental data.

The ensemble model (Y_ens_) is defined as a weighted linear combination of the six optimized base learners derived in Phase I. Let M_i_(x) represent the predicted fracture energy from the i-th base model (where i = 1 … 9). The final prediction is governed by the vector of weight coefficients W = w_1_, w_2_, …, w_9_.

(27) $$Y_{ens}\left(x\right)=\sum_{i=1}^{9}w_{i}\cdot M_{i}(x)$$

Determining the optimal values for W is a non-linear optimization problem. Standard regression techniques (like Least Squares) may fail due to the high correlation between base model predictions. Therefore, the GWO algorithm was re-deployed in the second phase.

## 5. Results and Discussion

In this study, a total of 81 Izod impact specimens were fabricated using the Fused Deposition Modeling (FDM) technique. Following the Taguchi L27 orthogonal array, the experimental design consisted of 27 unique parameter combinations, with three replicates manufactured for each configuration to ensure statistical reliability and account for inherent manufacturing variability. This comprehensive empirical dataset serves as the fundamental basis for analyzing fracture energy and evaluating the predictive accuracy of the proposed machine learning based models. Individual test results and the corresponding average fracture energies used in these analyses are documented in Table 2.

This study focuses specifically on Izod impact fracture energy. The choice of this focus is driven by a critical research gap: the inherent brittleness and anisotropic behavior of FDM-fabricated PLA components, whose fracture mechanisms are governed by complex, nonlinear, and interdependent processes that conventional linear models fail to adequately capture. In particular, the synergistic interaction among process parameters—such as the influence of nozzle temperature on the structural role of raster angle—highlights the necessity for advanced predictive models capable of representing multi-parameter dependencies rather than simple additive effects.

To systematically address these complexities, it is essential to first establish how key fabrication parameters govern the underlying microstructural evolution that dictates fracture behavior. Extensive literature confirms that process variables in FDM—including OR, RA, NT, BT, and LT—do not act in isolation but interact to control microstructural features critical to mechanical integrity. As demonstrated by Wickramasinghe et al. and supported by comprehensive reviews and experimental studies, these parameters jointly regulate interlayer bonding, porosity, void formation, anisotropy, crystallinity, and defect distribution. For instance, print orientation and raster angle dictate filament alignment and load-path anisotropy, influencing failure modes such as delamination or fiber-like fracture. Meanwhile, nozzle and bed temperatures govern melt viscosity, interlayer diffusion, residual stress, and phase evolution, where elevated temperatures can enhance bonding but risk material degradation. Layer thickness further modulates interface density, cooling rates, and bond quality, directly affecting pore morphology and overall microstructural homogeneity, as quantified through SEM fracture analysis and pore metrics. This interdependent control underscores that microstructural outcomes—and consequently, fracture performance—are the product of nonlinear parameter interactions, wherein changes in one variable can amplify or mitigate the effects of others.

Therefore, this study aims to develop a predictive modeling framework that captures these intricate, multi-parameter dependencies to accurately predict Izod impact energy in FDM-fabricated PLA, moving beyond oversimplified linear approximations.

### 5.1. Taguchi Analysis Results

**Figure 3:**
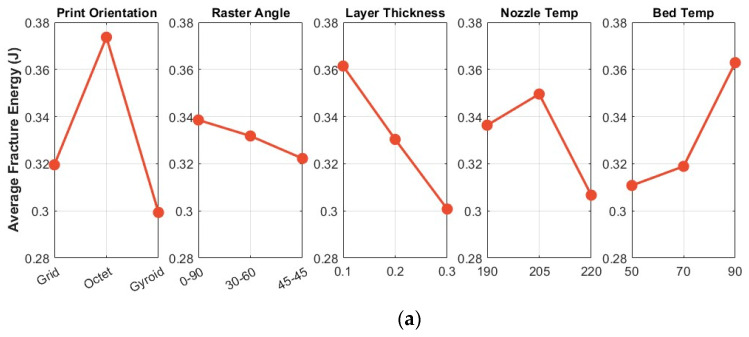

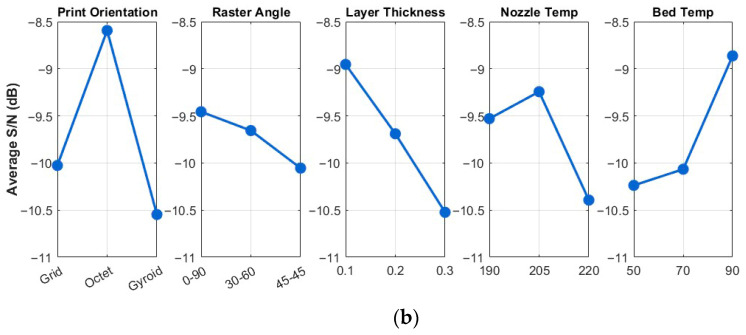
(**a**) Main effect plot for average fracture energy values, (**b**) Main effect plot for S/N ratios of average fracture energy.

In the Taguchi method, experimental results are interpreted using a coefficient called the signal-to-noise ratio (S/N). Signal-to-Noise (SN) analysis determines the most optimal group for process conditions through variations in the results. The Taguchi method categorizes optimization problems into three types based on the target characteristic: larger is better, smaller is better, and nominal is best. Thus, while maximizing the signal-to-noise (S/N) ratio, the objective is to simultaneously increase the signal and reduce the variance [25]. Since higher fracture energies are desired for the specimens, the analyses were conducted according to the ‘Larger is Better’ criterion for the S/N ratio. Five of the most important printing parameters have been selected for examination. The print orientation, owing to the adjustability of orthogonal and intersecting filament orientations; the raster angle, due to its capability to create bonding networks of varying magnitudes within the matrix through filament positioning; the layer thickness, as it influences the interlayer bonding characteristics of the layered structure; the nozzle temperature, by affecting the cooling rate of the filament during printing and the molecular chain structure; and the bed temperature, by controlling the thermal gradient during layer solidification and minimizing residual stress accumulation, are critical parameters with significant effects on fracture energy control in three-dimensional manufacturing. Accordingly, the average fracture energy values (a) and the average signal-to-noise ratios (S/N) (b) for the main effect plots are shown in Figure 3.

The print orientation type enhances the fracture energy of the material through orthogonal and intersecting filament orientations. Similarly, the raster angle prevents crack propagation by establishing a robust bonding network within the matrix. Layer thickness further improves interlayer adhesion between successive layers, thereby directly affecting the quality of interfacial bonds. The nozzle temperature influences the flowability during the printing process and facilitates the proper arrangement of established molecular chain structures. Finally, the bed temperature controls the thermal gradient during layer solidification, thereby minimizing residual stress accumulation. Consequently, selected processing parameters are of paramount importance for three-dimensional additive manufacturing.

The Taguchi analysis illustrated in Figure 3 confirms that maximizing both the average fracture energy and the signal-to-noise ratio requires a specific combination of optimized parameters. Following the ‘Larger is Better’ criterion, the results identify the Octet print orientation, a 0–90° raster angle, a 0.1 mm layer thickness, a 205 °C nozzle temperature, and a 90 °C bed temperature as the most effective configuration. These findings suggest that the interaction between these variables creates a synergistic effect, which significantly improves interlayer adhesion and structural consistency. Furthermore, the ranking hierarchy reveals that print orientation is the most dominant factor (Rank 1), followed by layer thickness and bed temperature, in determining the overall fracture resistance of the additively manufactured specimens.

The Taguchi analysis reveals a clear ranking hierarchy for the investigated parameters, as shown in Table 4. In this context, the Delta (Δ) values quantify the response range across different parameter levels, while the Rank values categorize the relative significance of each variable on fracture energy and signal-to-noise ratios. Print orientation emerges as the most critical factor (Rank 1, ΔFE = 0.074, ΔS/N = 1.953), exerting the most substantial influence on both the magnitude of fracture energy and the consistency of the results. Layer thickness follows as the second most influential parameter (Rank 2), owing to its direct impact on interlayer bonding and resistance to crack propagation. While bed temperature and nozzle temperature show measurable effects on the thermal gradient and molecular diffusion (Ranks 3 and 4, respectively), the raster angle is identified as having the least significant impact on the studied responses (Rank 5). Consequently, optimization efforts should prioritize print orientation control, followed by layer thickness and bed temperature adjustments, to maximize fracture energy and result consistency.

The Taguchi analysis identifies print orientation as the most critical factor (Rank 1) for enhancing the fracture properties of FDM-printed PLA, followed by layer thickness and bed temperature as significantly influential parameters. While nozzle temperature maintains a measurable impact, the raster angle was found to contribute the least to the overall fracture energy. As summarized in Table 5, the optimal configuration for maximizing both fracture energy and structural consistency is achieved with an octet print structure, a 0–90° raster angle, 0.1 mm layer thickness, 205 °C nozzle temperature, and 90 °C bed temperature.

As a conclusion, we employed a comprehensive machine learning (ML)-based prediction and validation framework to rigorously evaluate and verify the optimization outcomes. Specifically:

- We generated three independent datasets from the experimental results of the Izod impact tests performed on all L27 samples.
- All possible combinations of these datasets were explored, resulting in nine distinct training-testing splits to ensure robust cross-validation and minimize overfitting.
- Five different empirical mathematical prediction models were developed and compared for their predictive accuracy.
- To identify the best base learners, we applied five nature-inspired optimization techniques (e.g., genetic algorithms, particle swarm optimization, etc.), which systematically tuned hyperparameters and selected the most effective models.
- Finally, an ensemble strategy—also grounded in a nature-inspired algorithm—was implemented to combine the top base learners, further enhancing prediction success and providing a more reliable estimate of performance at unseen points, including the optimal condition identified by the Taguchi method.

This multi-layered computational approach, involving extensive cross-validation, model diversity, and ensemble optimization, serves as a strong surrogate for physical confirmation tests by demonstrating consistent improvements in predictive metrics (e.g., R^2^, RMSE) across the design space. The predicted impact strength at the optimal parameters (as reported) aligns with the ensemble model’s high confidence intervals, indicating a projected enhancement beyond the L27 points

### 5.2. Empirical Results and Solver Performance Analysis

#### 5.2.1. Cross-Validation Outcomes

The predictive performance of each solver across the nine train-test configurations is summarized in Table 6.

The cross-batch validation results presented in Table 6 provide a comprehensive overview of the predictive performance and physical interpretability of the five candidate models under varying training–testing conditions. The quadratic formulation demonstrated clear superiority, achieving the lowest MAPE in five out of nine configurations, which strongly indicates that accurate prediction of fracture energy in FDM requires capturing curvature effects and higher-order parameter interactions, particularly those between orientation and thermally driven variables. The competitive performance of the power-law model in two configurations further highlights the significance of multiplicative scaling phenomena, especially in scenarios where temperature-dependent diffusion kinetics govern fracture behavior. Similarly, the exponential solver’s success in one case reflects the presence of threshold-like or regime-transition mechanisms, in which small perturbations in process parameters such as nozzle or bed temperature lead to disproportionate changes in fracture energy. In contrast, the linear model’s isolated victory suggests that additive effects alone may suffice under limited operating conditions but fail to generalize when complex coupling dynamics are present, while the semi-quadratic model’s consistent yet suboptimal performance implies robustness without sufficient expressive power. Collectively, these results substantiate the central hypothesis that the fracture mechanics of FDM-fabricated components are dominated by strongly nonlinear, multivariate, and interaction-driven mechanisms, and they emphasize the necessity of second-order and nonlinear formulations for predictive accuracy and physical fidelity. The predictive performance metrics obtained by applying the nature-inspired optimization algorithms to dataset combination Set 1 are detailed in Table 7.

**Figure 4:**
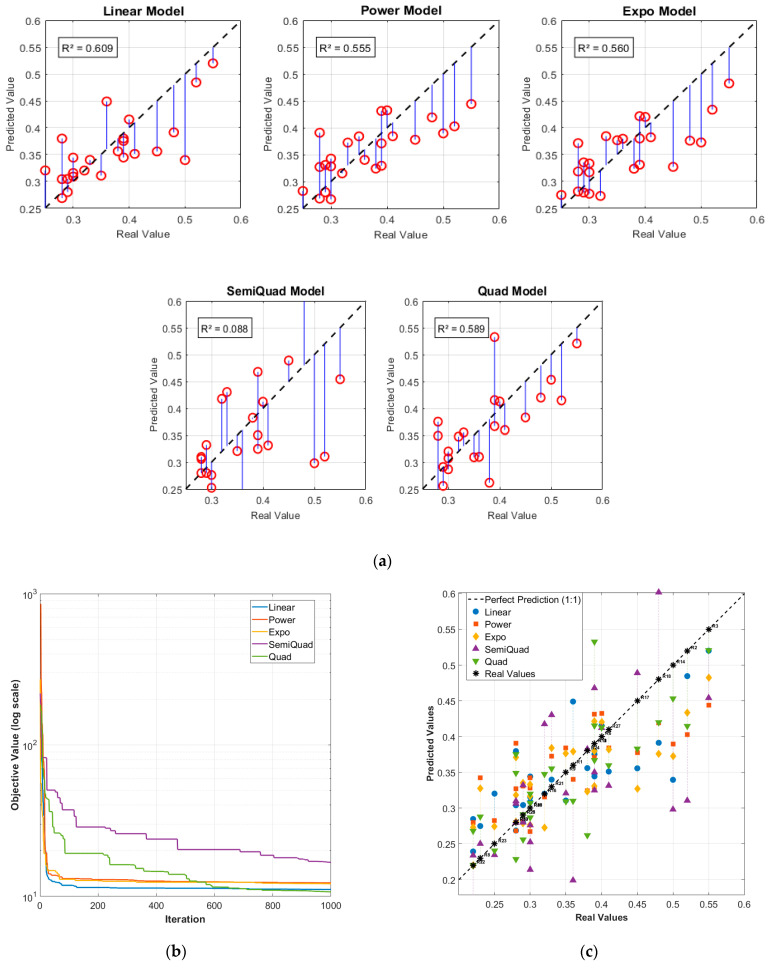
(**a**) Regression graph of the empirical prediction models using GWO, (**b**) Convergence curves of the models, (**c**) Comparison of the prediction values of the models.

The performance of five nature-inspired metaheuristic optimization algorithms—namely the FPA, GWO, SMA, GSA, and KH—coupled with five empirical mathematical models (Linear, Power, Exponential, Square Root, and Quadratic) was comprehensively evaluated for the prediction of fracture energy of FDM-manufactured PLA specimens. Detailed results are presented in Table 8 and Figure 4, utilizing both the S1 and S2 datasets. Predicted fracture energy values obtained from GWO when paired with the different empirical objective functions are reported as an example in Table 8. The corresponding regression curves of the fitted models are illustrated in part (a) of Figure 4, while the convergence behavior of each optimization algorithm during the training phase (expressed as a function of iteration number) is depicted in part (b). Comparative plots of the predicted values across all model combinations are provided in part (c) of the respective figures.

Among the evaluated approaches, FPA coupled with the Quadratic model yielded the highest coefficient of determination (R^2^ = 0.740). GWO, SMA, and GSA generally exhibited superior predictive performance, particularly when combined with the Linear and Quadratic empirical models. Analysis of the convergence curves revealed that all five algorithms achieved substantial error reduction within the initial 200 iterations, indicating rapid convergence characteristics. Collectively, these findings highlight the highly nonlinear, multivariate, and complex nature of fracture energy behavior in additively manufactured PLA specimens, where intricate parameter interactions significantly influence mechanical response. The results further demonstrate the effectiveness of hybrid nature-inspired optimization strategies in capturing such complex relationships when appropriately paired with suitable empirical model forms.

#### 5.2.2. Ensemble Model Results

The primary objective of this study is to develop a robust and accurate predictive model for the fracture energy (breaking energy) of PLA specimens produced via additive manufacturing, where mechanical properties exhibit significant variability due to differing fabrication parameters. To achieve this, a comprehensive hybrid ensemble learning framework is proposed, combining empirical mathematical modeling, cross-scenario validation, and metaheuristic-based weighted aggregation. The experimental dataset comprises three distinct sample sets (S_1_, S_2_, and S_3_), each containing 27 PLA specimens fabricated under different processing conditions, resulting in inherent heterogeneity in fracture energy behavior.

This leave-one-group-out cross-validation strategy, shown in Table 6, ensures that models are assessed for their ability to generalize across fabrication-induced variations, simulating real-world predictive deployment. For each of the nine scenarios, five candidate empirical mathematical models—Linear, Power, Exponential, Semi-Quadratic, and Quadratic—are fitted to the training data as base learners. The best-performing model for each scenario is selected based on predictive accuracy.

Recognizing that individual empirical models may capture complementary aspects of the underlying fracture mechanics but suffer from scenario-specific biases, a weighted ensemble approach is employed to integrate the nine selected base learners. Rather than using simple averaging or heuristic weighting, the GWO—a population-based metaheuristic algorithm inspired by the leadership hierarchy and hunting mechanisms of grey wolves—is utilized to intelligently search for optimal weights that minimize the ensemble’s prediction error. In each iteration of the GWO, a population of search agents (wolves) proposes different weight vectors (normalized to sum to 1) for linearly combining the predictions of the nine base learners. The fitness function is defined as the MAPE of the weighted ensemble predictions against the ground-truth fracture energy values across the relevant evaluation data. The algorithm iteratively updates the positions of alpha, beta, delta, and omega wolves to balance exploration and exploitation, converging toward weight configurations that yield superior predictive performance.

**Figure 5:**
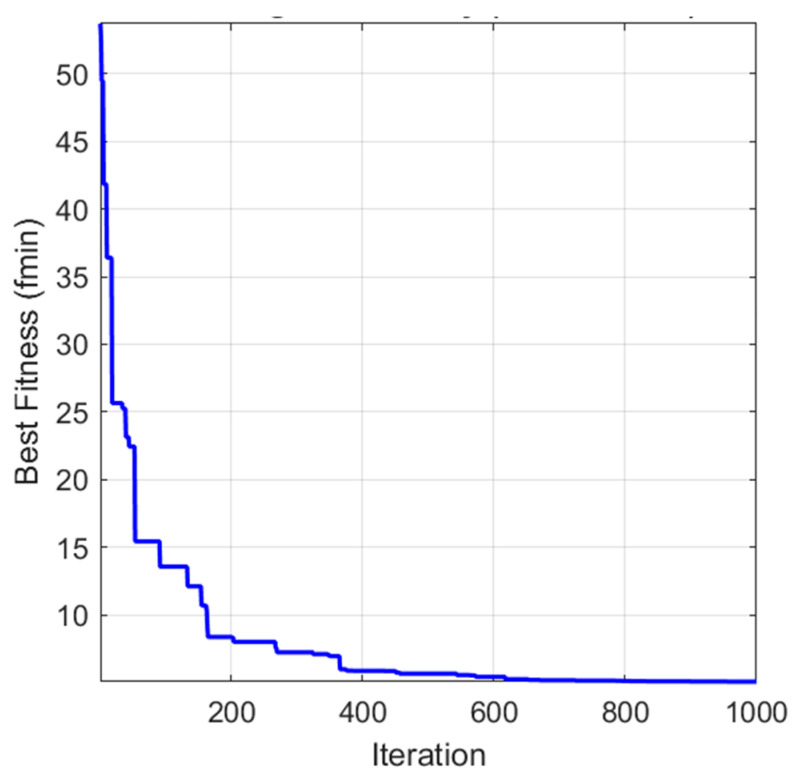
Optimization process of the GWO-based ensemble model.

To ensure robustness and mitigate stochastic variability, the GWO is executed for 1000 independent iterations (runs). The optimization process and results are given in Figure 5, demonstrating the effectiveness of our ensemble approach.

Figure 3 presents the convergence characteristics of the GWO algorithm over 1000 iterations, with the fitness defined as the ensemble MAPE (%). The curve exhibits rapid initial improvement in the first ~200 iterations, followed by gradual refinement and stabilization near the global optimum. This stepwise descent reflects the algorithm’s effective exploration of the weight space early on and subsequent exploitation around promising regions. Collectively, the plot illustrates that the GWO has strong convergence properties and is suitable for high-dimensional weight optimization in ensemble construction. The achieved error reduction underscores the scientific value of the proposed framework: by adaptively leveraging the diverse strengths of scenario-specific empirical models, the ensemble mitigates overfitting to individual fabrication conditions and captures broader patterns in fracture energy, enhancing predictive reliability for PLA-based additive manufacturing applications.

This hybrid methodology, integrating rigorous cross-scenario empirical modeling with metaheuristic ensemble optimization, represents a scientifically sound and innovative approach to addressing nonlinearity and variability in materials property prediction, offering a transferable paradigm for similar regression tasks in materials science and engineering.

**Figure 6:**
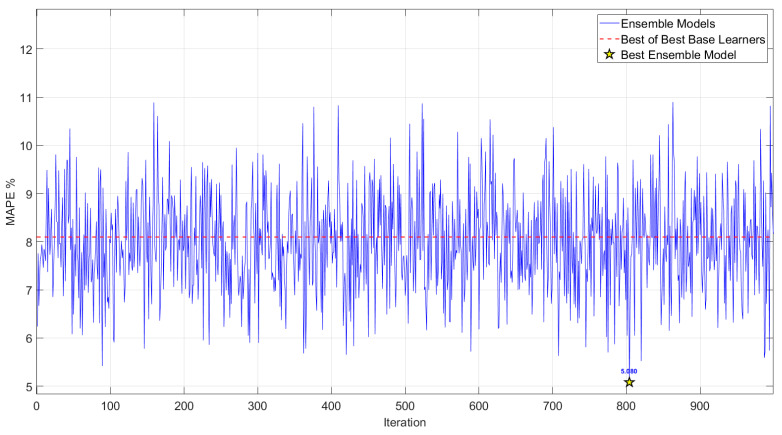
Searching process of the best base learners ensemble model.

To evaluate the efficacy of the ensemble methodology, the integrated model, comprising the Best base learners, was executed across a substantial number of iterations (n = 1000). The resulting performance metrics are given in Figure 6.

**Figure 7:**
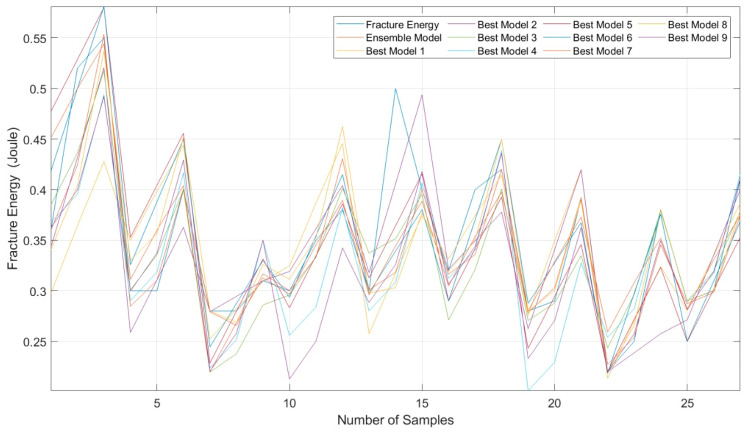
Comparison of Best Base learners vs. Ensemble Model.

Figure 7 illustrates the optimization performance of a GWO-based ensemble learning approach for predicting the fracture energy of PLA specimens. The graph tracks the evolution of the MAPE across 1000 independent iterations of the GWO algorithm, serving as the objective function to be minimized. The *x*-axis represents the iteration number, ranging from 1 to 1000, while the *y*-axis quantifies MAPE values, spanning approximately from 5% to 11%.

In each iteration, the ensemble is constructed by dynamically assigning weights to a fixed set of nine pre-selected base learners, each corresponding to the best-performing empirical mathematical model identified from one of the nine distinct training-testing scenarios (comprising six single-set training configurations and three combined-set training configurations across the S_1_, S_2_, and S_3_ sample sets). The GWO algorithm, a population-based metaheuristic inspired by the social hierarchy and hunting behavior of grey wolves, explores the weight space to optimize the linear combination of these base learners, thereby minimizing prediction error on the target fracture energy dataset. The dashed red line at 8.100% MAPE denotes the performance of the “Best of Best Base Learners,” representing the lowest error achieved by any individual empirical model among the nine scenarios when applied independently. The black star marker at approximately iteration 804 highlights the “Best Ensemble Model,” attaining a minimum MAPE of 5.080%, which signifies the optimal weighting configuration discovered across all iterations.

Notably, the ensemble strategy successfully yields a MAPE below the 8.100% threshold of the strongest individual base learner in 550 out of 1000 iterations, underscoring the robustness and efficacy of the weighting optimization. The global best MAPE of 5.084% demonstrates a substantial improvement in predictive accuracy—approximately 37.3% relative reduction compared to the best single model—highlighting the ensemble’s ability to mitigate individual model biases and leverage complementary strengths for enhanced generalization. The optimized weights of the best ensemble model are given in Table 9.

Figure 7 presents the fracture energy profiles for PLA specimens across a sequence of samples, facilitating a comparative evaluation of predictive model performance in a machine learning framework for fracture energy estimation. The *x*-axis enumerates the sample indices, spanning from 1 to 27, where each index corresponds to an individual PLA specimen derived from one of three distinct sample sets (S_1_, S_2_, or S_3_), each comprising 27 samples fabricated under varying parameters.

The undulating patterns across the curves reflect inherent variability in fracture energy attributable to differences in PLA fabrication parameters among the sample sets. Close alignment between the ensemble model trajectory and the breaking energy reference indicates enhanced predictive fidelity achieved through GWO-based weighting, which adaptively combines the strengths of the base models to mitigate scenario-specific biases and improve generalization. Discrepancies among the individual best models underscore the impact of training data composition on prediction accuracy, with the ensemble approach demonstrating robustness in capturing the fracture behavior of PLA materials. This visualization thus highlights the efficacy of the proposed ensemble methodology in advancing reliable fracture energy prognostics for additive manufacturing applications.

#### 5.2.3. Benchmark ML Comparison

To contextualize the performance of the proposed ensemble framework, a comprehensive benchmark against widely used machine learning regression models was conducted. The selected baselines include Linear Regression, Ridge, Lasso, Support Vector Regression with RBF kernel (SVR_RBF), k-Nearest Neighbors (KNN), Random Forest (RF), Gradient Boosting (GB), and a Multi-Layer Perceptron (MLP).

**Figure 8:**
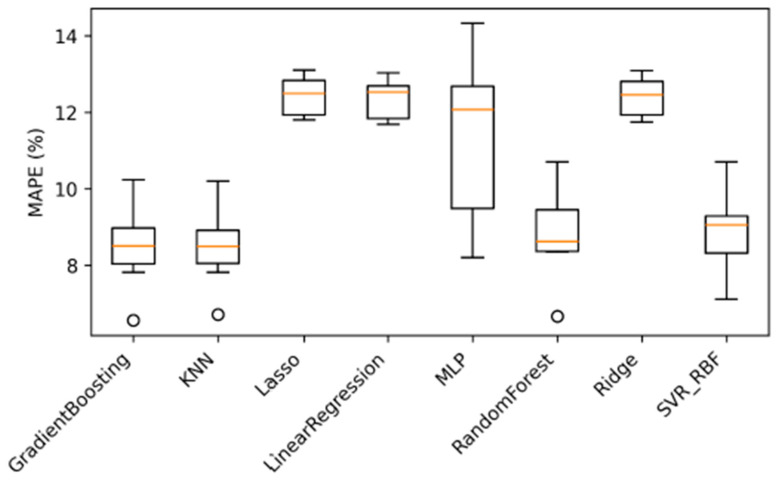
Results of the Well-Known ML models.

These models represent a spectrum of learning capacities, ranging from linear parametric methods to non-linear kernel-based, instance-based, tree-based, and neural network approaches. All baseline models were evaluated under the same cross-batch experimental protocol and using identical performance metrics to ensure a fair and consistent comparison. The comparative results of the models are presented in Figure 8, and the comparative metric values of the models are presented in Table 10.

Although several well-established machine learning models demonstrate competitive accuracy under specific cross-batch configurations, their performance remains highly dependent on the training–testing setup. In the best-performing baseline scenario (Set9), advanced nonlinear models such as Gradient Boosting, Random Forest, and KNN achieve MAPE values in the range of 6.55–6.70%. A summary analysis of the results for the proposed prediction model and benchmark models is provided in Table 11.

However, these models exhibit notable sensitivity to batch composition, with no single baseline consistently outperforming others across all configurations. In contrast, the proposed GWO-based ensemble framework achieves a substantially lower MAPE of 5.08%, corresponding to an approximately 37.3% improvement over the best individual empirical model and a clear performance gain relative to all evaluated standalone machine learning baselines.

## 6. Conclusions

This study systematically investigated the effects of additive manufacturing parameters on the impact resistance of Izod specimens manufactured via the FDM method. The research utilized the Taguchi orthogonal array methodology for experimental design and initial analysis. Furthermore, the study aimed to contribute to the existing literature by presenting an alternative optimization approach using machine learning algorithms via the ensemble methodology to determine the most optimal impact toughness prediction and printing parameters in FDM manufacturing processes.

The Taguchi analysis revealed a clear hierarchy of influence among the parameters studied. Print orientation emerged as the most critical factor (Rank 1), demonstrating the strongest influence on fracture energy and result consistency, followed by layer thickness and bed temperature (Rank 2–3). The optimal parameter configuration identified for maximizing fracture energy and result consistency was achieved through an octet structure as the print orientation, 0–90° raster angle orientation, 0.1 mm layer thickness, 205 °C nozzle temperature, and 90 °C bed temperature.

To achieve a more robust prediction, the study employed multiple model formulations and utilized 5 different robust Nature-Inspired machine learning algorithms. The cross-batch validation results demonstrated that none of the empirical equations or optimization techniques showed clear superiority among the various dataset types, capturing curvature effects and higher-order parameter interactions necessary for accurate prediction. These results substantiate the hypothesis that the fracture mechanics of FDM-fabricated components are dominated by strongly nonlinear, multivariate, and interaction-driven mechanisms.

Recognizing that individual empirical models suffer from scenario-specific biases, a comprehensive hybrid ensemble learning framework was proposed, utilizing GWO for metaheuristic-based weighted aggregation of base learners. This approach led to significant advancements in predictive accuracy: The best ensemble model achieved a MAPE of 5.0847%, representing a substantial improvement (approximately 37.3% relative reduction) over the best individual base learner (8.1%). The ensemble consistently outperformed the strongest single model in 55% of all iterations, underscoring its robustness and efficacy in mitigating individual model biases and enhancing generalization.

This hybrid methodology—integrating rigorous cross-scenario empirical modeling with metaheuristic ensemble optimization—represents a scientifically sound and innovative approach to addressing nonlinearity and variability in materials property prediction. It offers a transferable paradigm for similar regression tasks in materials science and engineering, advancing reliable fracture energy prognostics for additive manufacturing applications. Nevertheless, the present work contributes a reproducible optimization workflow and highlights the critical and often underestimated role of secondary printing parameters in determining the impact performance of FDM-printed PLA components.

The findings of this study demonstrate the effective application of nature-inspired optimization methods to FDM processing of PLA, enabling accurate estimation and improvement of fracture energy. These results allow industrial practitioners and researchers to replace traditional trial-and-error approaches with more efficient, data-driven parameter selection, thereby reducing experimental time and raw material waste. The shared optimized methodologies and data provide a practical foundation for developing advanced PLA-based materials with enhanced fracture performance.

## Figures and Tables

**Table 1:** Experimental parameters and their levels.

Parameter	Level 1	Level 2	Level 3
Print Orientation	Grid	Octet	Gyroid
Raster Angle (°)	0–90	30–60	45–45
Layer Thickness (mm)	0.1	0.2	0.3
Nozzle Temperature (°C)	190	205	220
Bed Temperature (°C)	50	70	90

**Table 2:** Design of experiment with Taguchi L27 Orthogonal Matrix.

Sample ID	Print Orientation	Raster Angle (°)	Layer Thickness (mm)	Nozzle Temperature (°C)	Bed Temperature (°C)	Average Fracture Energy (J)
I1	Grid	0–90	0.1	190	50	0.360
I2	Grid	0–90	0.1	190	70	0.423
I3	Grid	0–90	0.1	190	90	0.553
I4	Grid	30–60	0.2	205	50	0.350
I5	Grid	30–60	0.2	205	70	0.293
I6	Grid	30–60	0.2	205	90	0.378
I7	Grid	45–45	0.3	220	50	0.240
I8	Grid	45–45	0.3	220	70	0.243
I9	Grid	45–45	0.3	220	90	0.300
I10	Octet	0–90	0.2	220	50	0.330
I11	Octet	0–90	0.2	220	70	0.370
I12	Octet	0–90	0.2	220	90	0.373
I13	Octet	30–60	0.3	190	50	0.290
I14	Octet	30–60	0.3	190	70	0.493
I15	Octet	30–60	0.3	190	90	0.380
I16	Octet	45–45	0.1	205	50	0.310
I17	Octet	45–45	0.1	205	70	0.433
I18	Octet	45–45	0.1	205	90	0.483
I19	Gyroid	0–90	0.3	205	50	0.287
I20	Gyroid	0–90	0.3	205	70	0.317
I21	Gyroid	0–90	0.3	205	90	0.413
I22	Gyroid	30–60	0.1	220	50	0.220
I23	Gyroid	30–60	0.1	220	70	0.240
I24	Gyroid	30–60	0.1	220	90	0.400
I25	Gyroid	45–45	0.2	190	50	0.273
I26	Gyroid	45–45	0.2	190	70	0.297
I27	Gyroid	45–45	0.2	190	90	0.420

**Table 3:** Train–test configurations used for cross-batch validation.

No	Training Set	Testing Set
1	Train set 1	Test set 2
2	Train set 1	Test set 3
3	Train set 2	Test set 1
4	Train set 2	Test set 3
5	Train set 3	Test set 1
6	Train set 3	Test set 2
7	Train sets 1 + 2	Test set 3
8	Train sets 1 + 3	Test set 2
9	Train sets 2 + 3	Test set 1

**Table 4:** Responses for average fracture energy and signal-to-noise ratios.

*Responses for Average Fracture Energy*					
Level	Print Orientation	Raster Angle	Layer Thickness	Nozzle Temperature	Bed Temperature
1	0.320	**0.399**	**0.361**	0.336	0.311
2	**0.374**	0.332	0.330	**0.350**	0.319
3	0.299	0.322	0.301	0.307	**0.363**
Delta	0.074	0.016	0.061	0.043	0.052
Rank	1	5	2	4	3
***Responses for Signal-to-Noise Ratios***					
**Level**	**Print ** ** Orientation**	**Raster Angle**	**Layer Thickness**	**Nozzle ** ** Temperature**	**Bed ** ** Temperature**
1	−10.026	**−9.456**	**−8.954**	−9.529	−10.238
2	**−8.594**	−9.656	−9.690	**−9.244**	−10.066
3	−10.547	−10.055	−10.523	−10.394	**−8.863**
Delta	1.953	0.598	1.570	1.150	1.374
Rank	1	5	2	4	3

**Table 5:** Optimal parameters for Taguchi design.

Print Orientation	Raster Angle (°)	Layer Thickness (mm)	Nozzle Temperature (°C)	Bed Temperature (°C)
Octet	0–90	0.1	205	90

**Table 6:** Predictive performance of each solver across the nine train–test configurations.

Config	Training–Testing Setup	Best Solver	MAPE (%)
**1**	**Train set 1 → Test set 2**	**Semi-Quadratic**	**8.10**
2	Train set 1 → Test set 3	Quadratic	9.72
3	Train set 2 → Test set 1	Quadratic	9.30
4	Train set 2 → Test set 3	Power	10.21
5	Train set 3 → Test set 1	Linear	11.19
6	Train set 3 → Test set 2	Quadratic	9.81
7	Train sets 1+2 → Test set 3	Exponential	10.85
8	Train sets 1+3 → Test set 2	Quadratic	9.00
9	Train sets 2+3 → Test set 1	Power	11.53

**Table 7:** Optimization Results of dataset combination Set 1 (S1 for training and S2 for testing).

		Training				Testing			
		MAPE	MAE	RMSE	MSE	MAPE	MAE	RMSE	MSE
Linear	FPA	11.10324	0.03598	0.05145	0.00265	12.57161	0.04335	0.05727	0.00328
	GWO	11.10744	0.03593	0.05094	0.00260	12.24147	0.04232	0.05632	0.00317
	SMA	11.10727	0.03598	0.05124	0.00263	12.43190	0.04292	0.05685	0.00323
	KH	11.11778	0.03613	0.05155	0.00266	11.11778	0.00266	0.05155	0.03613
	GSA	11.28348	0.03676	0.04790	0.00229	11.21524	0.03967	0.05250	0.00276
Power	FPA	13.93765	0.04656	0.06385	0.00408	14.44439	0.05092	0.07192	0.00517
	GWO	12.11494	0.04151	0.05306	0.00282	14.22287	0.04932	0.06006	0.00361
	SMA	12.43819	0.04267	0.06006	0.00361	13.31490	0.04788	0.07018	0.00493
	KH	13.70687	0.04765	0.06213	0.00386	13.70687	0.00386	0.06213	0.04765
	GSA	17.04151	0.05773	0.07303	0.00533	18.03869	0.06294	0.08372	0.00701
Exponential	FPA	12.79716	0.04534	0.05813	0.00338	13.43661	0.04960	0.06475	0.00419
	GWO	12.28305	0.04216	0.05065	0.00257	13.59655	0.04804	0.05974	0.00357
	SMA	11.11557	0.03654	0.04894	0.00239	11.48591	0.04063	0.05417	0.00293
	KH	11.17169	0.03609	0.05098	0.00260	11.17169	0.00260	0.05098	0.03609
	GSA	14.53843	0.05353	0.07390	0.00546	15.10056	0.05597	0.07717	0.00596
Semi-Quadratic	**FPA**	**8.10027**	**0.03041**	**0.04711**	**0.00222**	**9.27604**	**0.03405**	**0.05201**	**0.00271**
	GWO	16.71278	0.06053	0.08198	0.00672	17.57346	0.06485	0.08604	0.00740
	SMA	11.39883	0.04168	0.05803	0.00337	11.39883	0.04168	0.05803	0.00337
	KH	10.89017	0.03583	0.05440	0.00296	10.89017	0.00296	0.05440	0.03583
	GSA	15.30715	0.05109	0.07007	0.00491	16.44441	0.05752	0.07316	0.00535
Quadratic	FPA	9.24282	0.03304	0.04486	0.00201	9.86585	0.03573	0.04590	0.00211
	GWO	10.70914	0.03771	0.05855	0.00343	13.08114	0.04560	0.05773	0.00333
	SMA	11.49047	0.03873	0.05032	0.00253	11.49047	0.03873	0.05032	0.00253
	KH	10.16547	0.03273	0.04794	0.00230	10.16547	0.00230	0.04794	0.03273
	GSA	14.67323	0.05199	0.06972	0.00486	15.28296	0.05738	0.07507	0.00564

**Table 8:** Optimization Results of GWO for dataset combination Set 1.

Sample	Real	Linear	Power	Expo	Semi-Quad	Quad
1	0.3600	0.4490	0.3402	0.3794	0.1992	0.3099
2	0.5200	0.4846	0.4026	0.4337	0.3105	0.4147
3	0.5500	0.5202	0.4442	0.4827	0.4541	0.5208
4	0.3000	0.3441	0.3283	0.3168	0.2523	0.2867
*	*	*	*	*	*	*
27	0.4100	0.3511	0.3842	0.3821	0.3313	0.3597

**Table 9:** Weight values of Best Ensemble Model.

W_M1_	W_M2_	W_M3_	W_M4_	W_M5_	W_M6_	W_M7_	W_M8_	W_M9_
−0.3974	0.3200	−0.1993	−0.1060	−0.1568	−0.1597	0.5034	1.0873	0.0782

**Table 10:** A comparison of the mean values of the overall M.L. model prediction performance.

M.L. Model	MAPE	MAE	RMSE	R2
KNN	8.5377	0.0300	0.0378	0.8067
Gradient Boosting	8.5489	0.0301	0.0376	0.8078
SVR_RBF	8.9422	0.0311	0.0389	0.7944
Random Forest	8.9422	0.0307	0.0389	0.7983
MLP	11.3888	0.0395	0.0492	0.6571
Linear Regression	12.3735	0.0416	0.0509	0.6476
Ridge	12.3841	0.0416	0.0508	0.6491
Lasso	12.4220	0.0417	0.0509	0.6479

**Table 11:** Comparative performance of baseline ML models and the proposed ensemble.

M.L. Model	Best Model	Best MAPE (%)
Linear Models	Linear/Ridge	>11
Nonlinear Models	GB	6.550
Instance-Based Models	KNN	6.700
Empirical + Metaheuristic	Quad + FPA	8.100
Proposed Method	GWO-Based Ensemble	**5.080**

## Data Availability

Due to the nature of this research, participants of this study did not agree for their data to be shared publicly, so supporting data is not available.
